# Tuftelin 1 (TUFT1) Promotes the Proliferation and Migration of Renal Cell Carcinoma *via* PI3K/AKT Signaling Pathway

**DOI:** 10.3389/pore.2021.640936

**Published:** 2021-04-19

**Authors:** Hua Lin, Weifeng Zeng, Yuhang Lei, Desheng Chen, Zhen Nie

**Affiliations:** Department of Urology, Xiantao First People’s Hospital Affiliated to Yangtze University, Xiantao, China

**Keywords:** renal cell carcinoma, tuftelin 1, proliferation, migration, signaling pathway

## Abstract

Tuftelin 1 (TUFT1), a protein functioning distinctively in different tissues, is reported to be elevated in several types of cancers and the elevation of TUFT1 is correlated with unfavorable clinicopathologic characteristics and poor survival. However, the involvement of TUFT1 in renal cell carcinoma (RCC) remains unknown. In the current study, we investigated the role of TUFT1 in RCC and potential underlying mechanisms. RT-PCR and Western blot analysis showed that both the mRNA and protein levels of TUFT1 were increased in primary RCC tissue and RCC cell lines. TUFT1 overexpression in RCC cells resulted in enhanced cell proliferation and migration while knockdown of TUFT1 by contrast decreased the growth and migration of the RCC cells, indicating TUFT1 expression is involved in RCC cell growth and migration. The involvement of TUFT1 in the epithelial-mesenchymal transition (EMT) of RCC cells was also determined by measuring the expression of EMT-related markers. Our data showed that TUFT1 overexpression promoted RCC cell EMT progression while knockdown of TUFT1 suppressed such process. Further signaling pathway inhibition assay revealed that TUFT1-induced RCC cell growth, migration and EMT was significantly suppressed by PI3K inhibitor, but not JNK or MEK inhibitors. In addition, TUFT1 overexpression enhanced the AKT phosphorylation, a key member of the PI3K signaling pathway, while PI3K inhibitor suppressed such process. Taken together, our study showed that TUFT1 expression was elevated in RCC and such elevation promoted the proliferation, migration and EMT of RCC cells *in vitro*, through PI3K/AKT signaling pathway. The findings of our current study imply that TUFT1 is involved in RCC tumorigenesis, and it may serve as a biomarker for RCC diagnosis and a potential target for RCC treatment.

## Introduction

Renal cell carcinoma (RCC), with about 403,000 newly diagnosed cases and around 175,000 deaths annually, is one of the most common cancers worldwide [1]. RCC represents a heterogeneous group of cancers that are derived from renal tubular epithelial cells [2]. Although remarkable advances have been achieved in the understanding, diagnosis and treatment of this disease, the prognosis of RCC patients still remain unsatisfactory. Up to now, surgical removal still remains as the only curative approach [3–5]. For patients with advanced RCC, systemic immunomodulatory therapies using drugs like interferon-α, IL-2, sunitinib and sorafenib have been possible [6–9]. Recently, the therapeutic spectrum has been broadened by several novel targeted agents, including as VEGF and mTOR inhibitors and the programmed cell death-1 (PD-1) immune checkpoint inhibitors (nivolumab, pembrolizumab and avelumab) [10–13]. Such immunomodulatory therapies, especially the targeted therapies have significantly improved objective response rate and/or median progression-free survival with reduced the adverse effects [7, 8, 14]. Despite these advances, tumor long-term responsiveness to these drugs are poor and patient survival rate is only slightly increased [15]. Therefore, the development of new drugs with better efficacy is still in need, while understanding the mechanism underlying RCC tumorigenesis would significantly benefit drug development.

Tuftelin 1 (TUFT1), a phosphorylated glycoprotein initially found in tooth enamel, plays a role in dental enamel mineralization [16]. The expression of TUFT1 is later also found in other tissues and the function of TUFT1 seems to be tissue dependent [17]. Recently, TUFT1 expression is reported to be elevated in several types of cancers including hepatocellular carcinoma (HCC), breast cancer, thyroid carcinoma and osteosarcoma [18–20]. Increased expression of TUFT1 can promote the growth and metastasis of cancer cells through distinctive pathways in different cancers [20, 21]. Moreover, TUFT1 expression is associated with unfavorable clinical outcomes and poor prognosis of HCC [20]. However, whether TUFT1 also plays a role in RCC still remains to determined.

In the current study, we demonstrated that TUFT1 expression was also increased in RCC tissue and cell lines. Further mechanistic investigation *in vitro* showed that TUFT1 promoted RCC cell proliferation, migration, and epithelial-mesenchymal transition (EMT), through PI3K/AKT signaling pathway. The findings of our current study imply that TUFT1 is involved in RCC tumorigenesis, and it may serve as a biomarker for RCC diagnosis and a potential target for RCC treatment.

## Materials and Methods

### Ethical Statement and Patient Samples

All protocols involving human specimens were reviewed and approved by the Ethics Review Board at Xiantao First People’s Hospital (No. 2018H00109). Written informed consents were obtained from all participants. RCC tissues (CT) and adjacent noncancerous tissues (NT) were obtained from 10 RCC patients who underwent surgical resection at Xiantao First People’s Hospital. None of the 10 patients received any anti-RCC treatment before the surgery. The clinicopathological data of the patients were listed in Supplementary Table S1. Harvested tissue samples were immediately stored at −80°C till use.

### Cells and Plasmids

Human normal renal proximal tubule epithelial cells (RPTEC), RCC cell lines A498 and 786-O were all purchased from ATCC, and cultured in DMEM/F12, EMEM and RPMI-1640 medium, respectively. A final concentration of 10% FBS (Gibco, Thermo Fisher Scientific) and antibiotics were added to all growth media. The human TUFT1 expression plasmid, designated as pTUFT1, was a kind gift from Dr. Kai Hu from Wuhan Institute of Virology, Chinese Academy of Sciences. TUFT1 shRNA (cat no: sc-61736-SH) and control shRNA (cat no: sc-108060) were both purchased from Santa Cruz Biotechnology.

### RT-PCR

RT-PCR was performed as previously described with modifications [18, 22]. In brief, total RNA was extracted using Trizol (Thermo Fisher Scientific) and reverse-transcribed into cDNA with FastQuant RT Kit (TIANGEN), according to the manufacturer’s instructions. TUFT1 mRNA level was then semi-quantified using a SYBR Green-based RT-PCR. GAPDH was used as an internal control. The primer pair used for TUFT1 detection were (forward) 5′-TCA​GAC​TGT​GTG​GCT​TTT​GAG-3′ and (reverse) 5′-GTC​AGC​ATT​GTT​GCT​CCG​AAG-3′. The primer pair used for GAPDH detection were (forward) 5′- GCC​AAG​GTC​ATC​CAT​GAC​AAC​TTT​GG-3′ and (reverse) 5′- GCC​TGC​TTC​ACC​ACC​TTC​TTG​ATG​TC-3′. The relative expression of TUFT1 was calculated using the 2^−ΔΔCt^ method.

### Transfection and Inhibitor Treatment

All transfections were conducted using Lipo8000™ Transfection Reagent (Beyotime) according to the manufacturer’s instructions. In brief, plasmids were first diluted in Opti-MEM (Thermo Fisher Scientific) and then mixed with Lipo8000™ Transfection Reagent. Afterwards, the prepared the mixture was directly added into cell culture and cultured for 48 h. For signaling pathway inhibitor treatment, transfection mixture was removed 4 h post transfection and fresh medium supplemented with inhibitors were added and cells were cultured in the presence of inhibitor for another 48 h. The following signaling pathway inhibitors were used in the current study: SP600125 (JNK inhibitor; 5 μM), LY294002 (PI3K inhibitor; 25 μM) and PD98059 (MEK inhibitor; 50 μM). All inhibitors were purchased from Selleck and used as per the manufacturer′s instructions.

### Western Blot

Western blot was performed as previously described with modifications [20, 23]. In brief, cells were first lysed using lysis buffer (150 mM NaCl, 1% NP-40 and 50 mM Tris pH 8.0) supplemented with protease inhibitor cocktail (Beyotime), and then cleared cell lysates were mixed with SDS-PAGE loading buffer (50 mM Tris-HCl, pH 6.8, 2% SDS, 25% glycerol) and boiled for 10 min. Prepared samples were then separated by a 12% SDS-PAGE gel and transferred onto a PVDF membrane. The membrane was subsequently blocked with 5% non-fat milk for 1 h at room temperature. After three washes with PBST, membrane was then sequentially incubated with primary antibodies and HRP-conjugated secondary antibodies overnight at 4°C and 1 h at room temperature, respectively. Following extensive washes, membrane was developed with ECL substrate (Beyotime), according to the manufacturer’s instructions. The following primary antibodies were used: rabbit anti-human TUFT1 antibody (1:1,000 dilution; Cat no: 23385-1-AP; Proteintech), mouse anti-human E-cadherin antibody (1:1,000 dilution; Cat no: 60335-1-Ig; Proteintech), mouse anti-human N-cadherin antibody (1:1,000 dilution; Cat no: 66219-1-Ig; Proteintech), rabbit anti-human Snail (1:1,000 dilution; Cat no: 13099-1-AP; Proteintech), mouse anti-human AKT antibody (1:1,000 dilution; Cat no: 60203-2-Ig; Proteintech), rabbit anti-human phospho-AKT (Thr308) (*p*-AKT (Thr308), 1:1,000 dilution; Cat no: 9275; Cell Signaling Technology), mouse anti-human phospho-AKT (Ser473) (*p*-AKT (Ser473), 1:1,000 dilution; Cat no: 66444-1-Ig; Proteintech) and mouse anti-human GAPDH (1:2,000 dilution; Cat no: A00227; Boster). The following secondary antibodies were used: HRP-conjugated goat anti-mouse IgG (H + L) (1:30,000 dilution; Cat no: BA1050; Boster) and HRP-conjugated goat anti-rabbit IgG (H + L) (1:30,000 dilution; Cat no: BA1054; Boster). Western blot band intensity was semiquantified by ImageJ (version 1.53c; National Institutes of Health, Bethesda, MD, United States) and relative protein expression was calculated using GAPDH as the reference.

### MTT Assay

The cell proliferation was assessed using a MTT cell proliferation and cytotoxicity assay kit (Sangon Biotech), according to the manufacturer’s instructions. In brief, cell culture medium was removed, and cells were treated with 1:1 diluted MTT solution in fresh serum-free medium for 3 h at 37°C. Following incubation, MTT solvent was added into each well and incubated on an orbital shaker for 15 min. Finally, the samples were read at OD590 nm with a microplate reader.

### Wound Healing Assay

Cell migration was determined by wound healing assay, as previously described with modifications [24, 25]. In brief, cells pre-seeded at 50–60% confluency were first transfected with vector, pTUFT1, control shRNA or TUFT1 shRNA, and when cells formed a monolayer, a “wound” was introduced by scratching the cell monolayer with a sterile 200 µl tip. After washes with PBS to remove non-attached cells, the cell monolayer was then incubated in fresh medium and cultured for 24 h. The cell migration was measured by the “wound healing” rate. For cells receiving inhibitor treatment, inhibitors or mock controls were introduced into the culture right after the PBS washes.

### Statistical Analysis

All data were expressed as mean ± standard deviation (SD), and all statistical analyses were performed with Prism 8 (GraphPad). Either a Mann-Whitney test or a Kruskal-Wallis test with Dunn’s multiple comparisons test were used, and a *p* value less than 0.05 was considered statistically significant.

## Results

### TUFT1 Expression Is Elevated in RCC Tissues and Cell Lines

**FIGURE 1:**
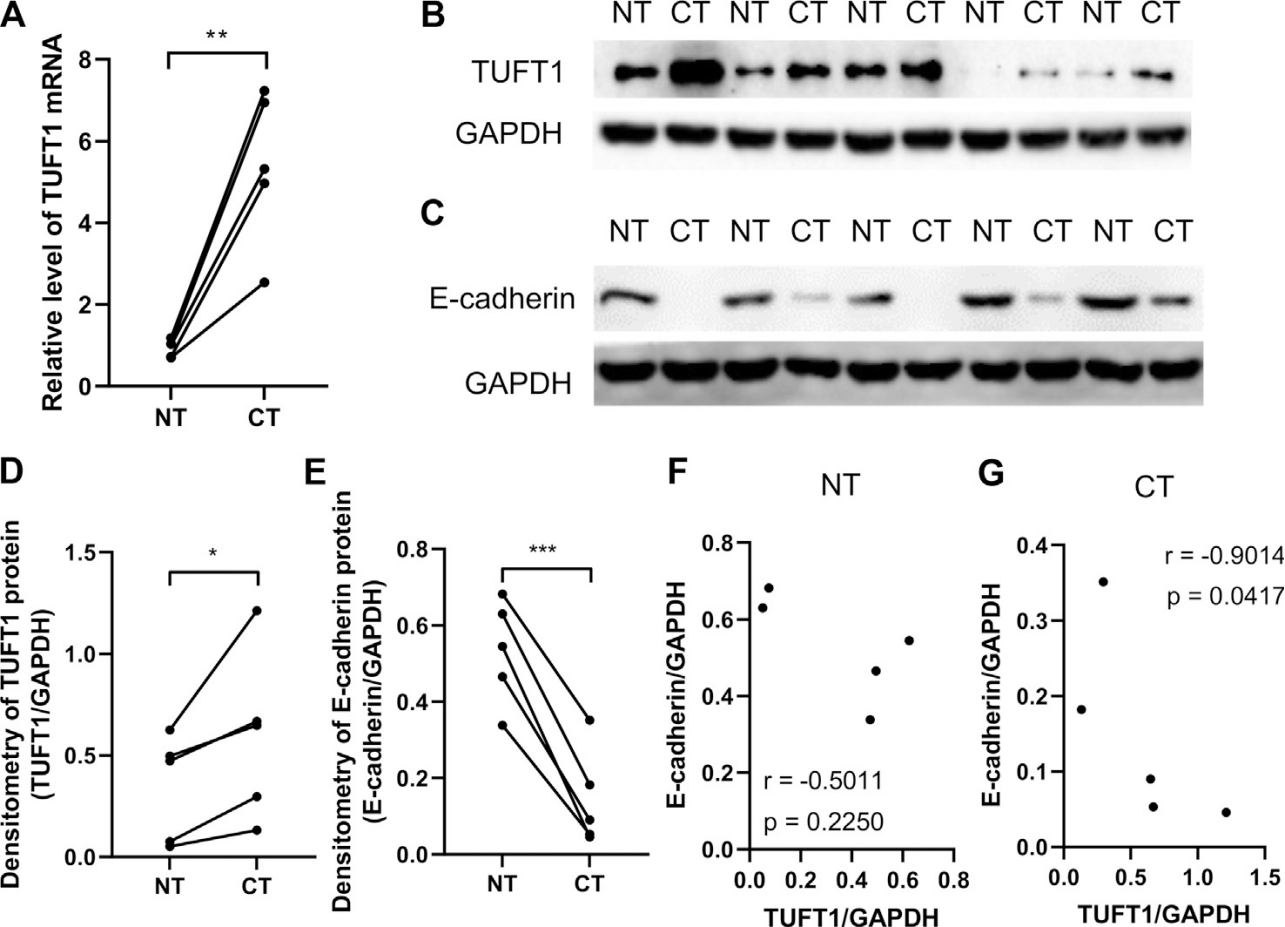
TUFT1 expression is elevated in RCC tissue. Cancerous tissue (CT) and adjacent noncancerous tissue (NT) were collected from RCC patients and **(A)** the mRNA and **(B, D)** protein level of TUFT1 and **(C, E)** E-cadherin were determined by RT-PCR and Western blot, respectively (*n* = 5). **(F, G)** The correlation between TUFT1 and E-cadherin expression in **(F)** NT samples and **(G)** CT samples was analyzed with Spearman correlation analysis. *, *p* < 0.05; **, *p* < 0.01; ***, *p* < 0.001.

TUFT1 expression was previously reported to be elevated in HCC cancer tissue (CT) comparing to adjacent noncancerous tissue (NT) [20]. To check if a similar trend could be observed in RCC, we collected 10 pairs of CT and NT samples from RCC patients who underwent surgical resection and measured the TUFT1 mRNA and protein levels. To rule out possible drug interference, none of the patients had received any anti-RCC treatment before surgery. Our data showed similar results to what was reported in HCC (Figures 1A,B,D and Supplementary Figure S1). Namely, both the mRNA and protein levels of TUFT1 was significantly increased in CT comparing to adjacent NT.

**FIGURE 2:**
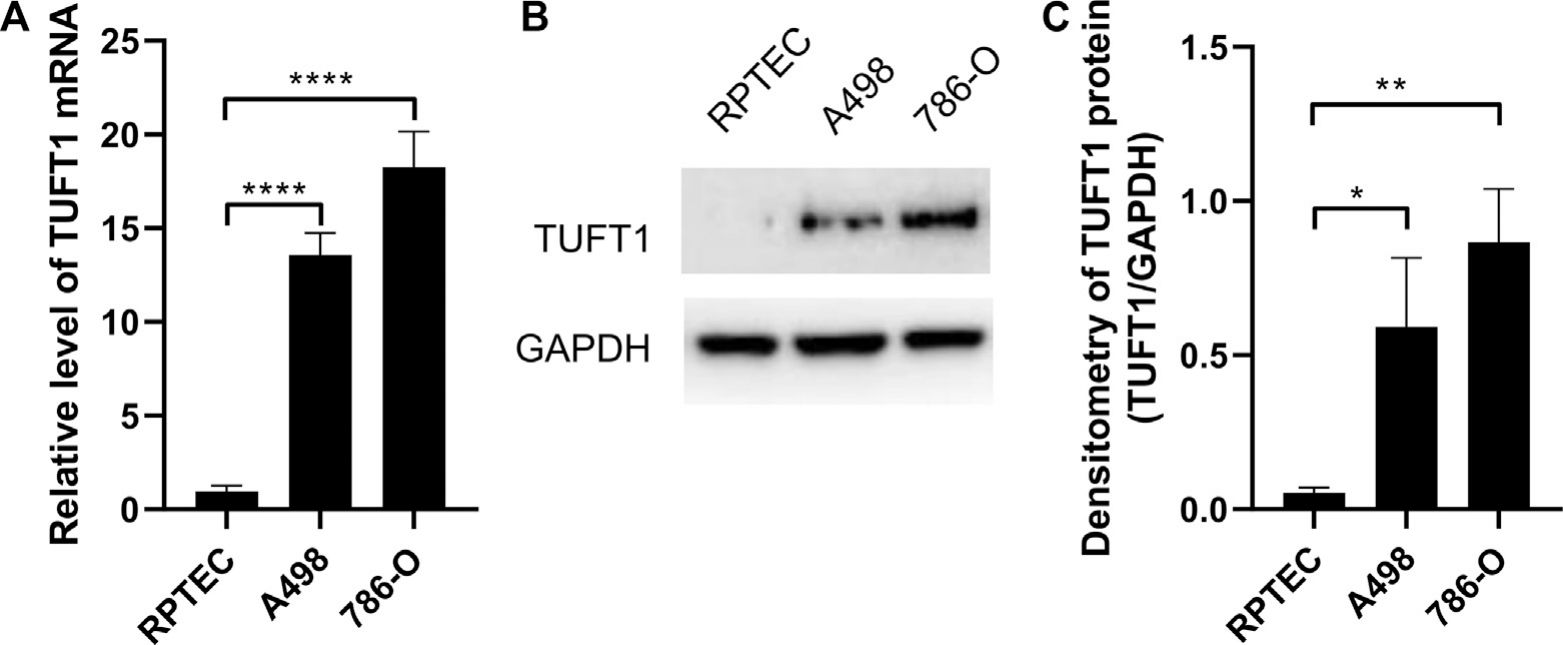
TUFT1 expression is elevated in RCC cell lines. **(A)** The mRNA and **(B, C)** protein level of TUFT1 in normal renal epithelial cells (RPTEC) and RCC cell lines A498 and 786-O were determined by RT-PCR and Western blot, respectively. **(A)** Data shown are mean ± SD of three independent experiments. **(B)** One representative result is shown. **(C)** Band intensity of **(B)** was semi-quantified by Image J. *, *p* < 0.05; **, *p* < 0.01; ****, *p* < 0.0001.

We next further investigated whether such elevation in TUFT1 expression could also be observed in RCC cell lines. TUFT1 mRNA level in one normal renal proximal tubule epithelial cells (RPTEC) and 2 RCC cell lines A498 and 786-O were measured by RT-PCR. Consistently, TUFT1 mRNA was significantly increased in both RCC cell lines (Figure 2A). Similar results were observed when the TUFT1 protein level was determined by Western blot (Figures 2B,C). Taken together, our data here indicate that TUFT1 expression is upregulated in RCC.

### TUFT1 Promotes RCC Cell Growth and Migration

**FIGURE 3:**
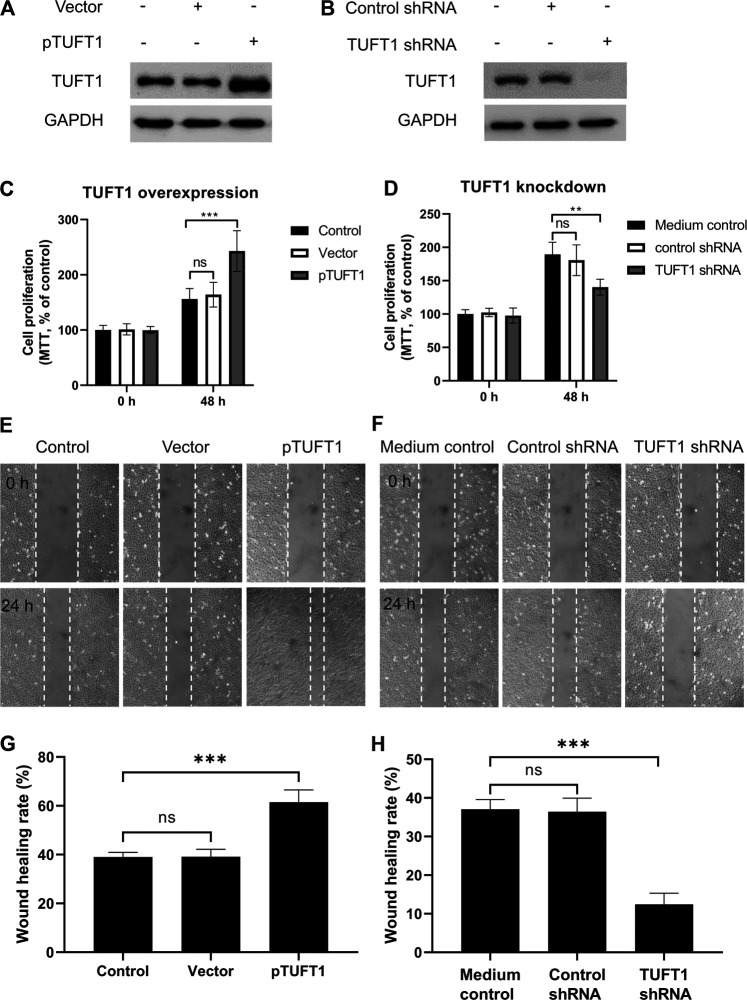
TUFT1 promotes RCC cell growth and migration. A498 cells were either transfected with **(A, C, E, G)** pTUFT1 or **(B, D, F, H)** TUFT1 shRNA, and then **(A**, **B)** TUFT1 expression was determined by Western blot, **(C, D)** cell proliferation was measured by MTT assay and **(E–H)** cell migration was determined by wound healing assay. **(A, B, E, F)** One representative result is shown. **(C, D, G, H)** Data shown are mean ± SD of three independent experiments. Ns, not statistically significant; **, *p* < 0.01; ***, *p* < 0.001.

To investigate the impact of TUFT1 expression on RCC cell growth and migration, TUFT1 expression was either upregulated or downregulated in A498 cells, and then cell proliferation and migration were determined. As shown in Figure 3A and Supplementary Figure S2, transfection of pTUFT1 considerably increased TUFT1 protein expression in A498 cells. In accordance with the increased TUFT1 expression, the proliferation rate of the A498 cells with TUFT1 overexpression showed significantly enhanced cell growth (Figure 3C) and migration (Figures 3E,G), comparing to vector-transfected cells. By contrast, cells with TUFT1 shRNA transfection showed downregulated TUFT1 expression (Figure 3B). Accordingly, cells with decreased TUFT1 showed slower proliferation rate (Figure 3D) as well as slower migration (Figures 3F,H). Together, these data indicate that TUFT1 enhances RCC cell growth and migration.

### TUFT1 Promotes the Epithelial-Mesenchymal Transition (EMT) of RCC Cells

**FIGURE 4:**
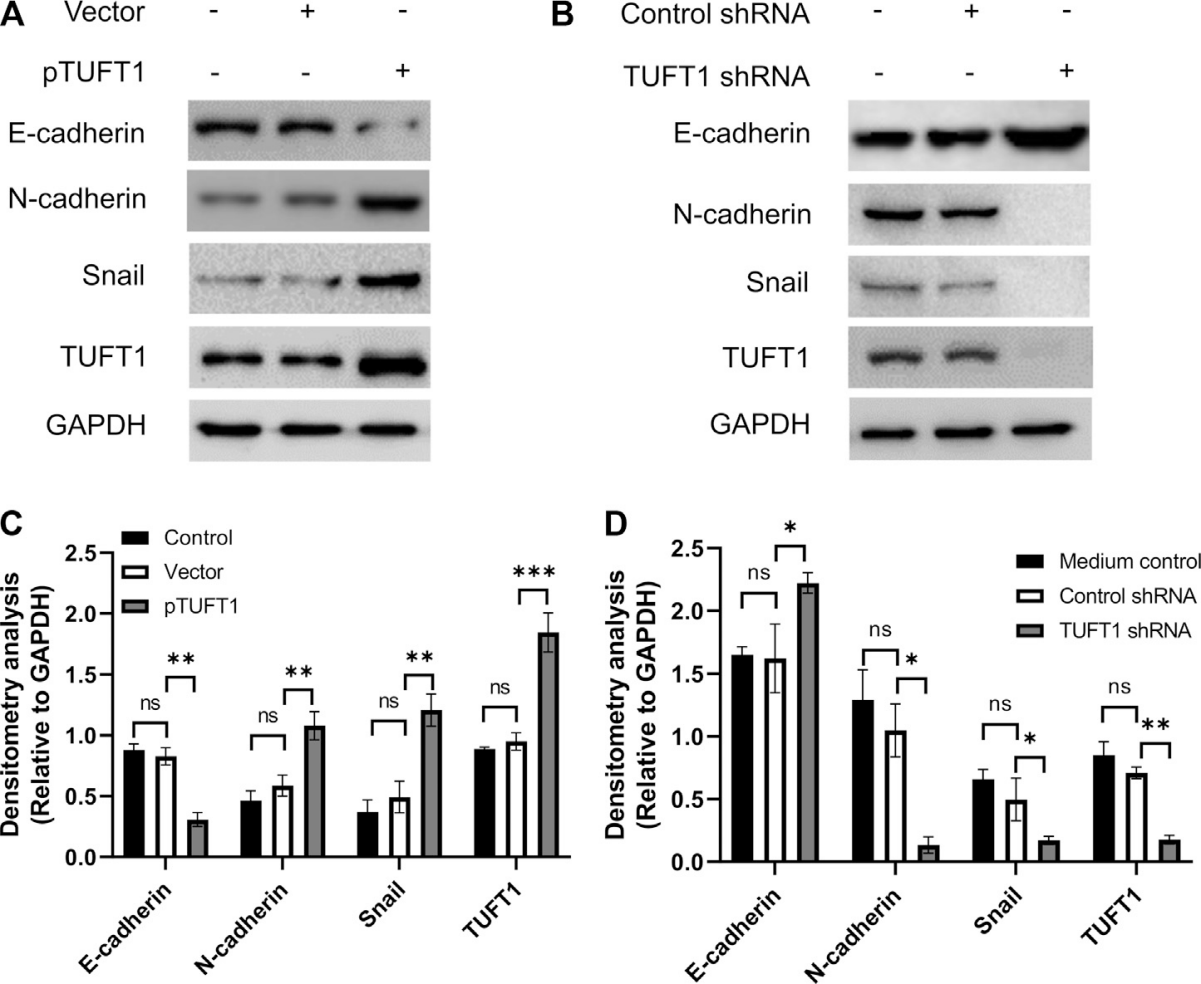
TUFT1 promotes the EMT of RCC cells. A498 cells were first transfected with either **(A)** pTUFT1 or **(B)** TUFT1 shRNA, and then the expression of E-cadherin, N-cadherin, Snail and TUFT1 was measured by Western blot. One representative result is shown. **(C, D)** Band intensity analysis of **(A)** and **(B)**, respectively. Data shown are mean ± SD of three independent experiments. Ns, not statistically significant; *, *p* < 0.05; **, *p* < 0.01; ***, *p* < 0.001.

EMT is a biological process allowing immobilized polarized epithelial cells transdifferentiate into motile mesenchymal cells, which exhibit enhanced invasiveness and resistance to apoptosis [26, 27]. The activation of an EMT process is considered the critical mechanism mediating cancer metastasis [28]. The involvement of TUFT1 in the regulation of EMT in RCC cells were subsequently investigated by determining the expression of EMT-related markers (E-cadherin, N-cadherin and Snail). E-cadherin is an important adhesion molecule maintaining the epithelial phenotype while loss of E-cadherin leads to enhanced cell mobilization and activation of several EMT transcription factors [29]. Decrease of E-cadherin is considered the EMT hallmark. N-cadherin, in contrast, is mainly expressed in non-epithelial cells and serves as an indicator of ongoing EMT [30, 31]. Snail is one of the essential EMT-inducing transcription factors that facilitates a mesenchymal phenotype [32]. Our data showed that in A498 cells with TUFT1 overexpression, the expression of E-cadherin was decreased while the expression of N-cadherin and Snail was increased, indicating that TUFT1 promoted EMT of RCC cells (Figures 4A,C). To further confirm that TUFT1 is involved in EMT of RCC cells, a downregulation of TUFT1 in A498 cells was performed by TUFT1 shRNA. By contrast to TUFT1 overexpression, elevation of E-cadherin expression while decrease of N-cadherin and Snail were observed in TUFT1 knockdown cells (Figures 4B,D). In accordance with the cell line data, E-cadherin expression was decreased in primary RCC tumor tissue than in adjacent noncancerous tissue (Figures 1C,E). Furthermore, a negative correlation between TUFT1 and E-cadherin expression was detected in the CT but not NT clinical samples (Figures 1F,G). Together, our data demonstrate that TUFT1 promotes EMT progression of RCC cells.

### TUFT1 Exerts Oncogenic Effects on RCC Cells Through PI3K/AKT Signaling Pathway

**FIGURE 5:**
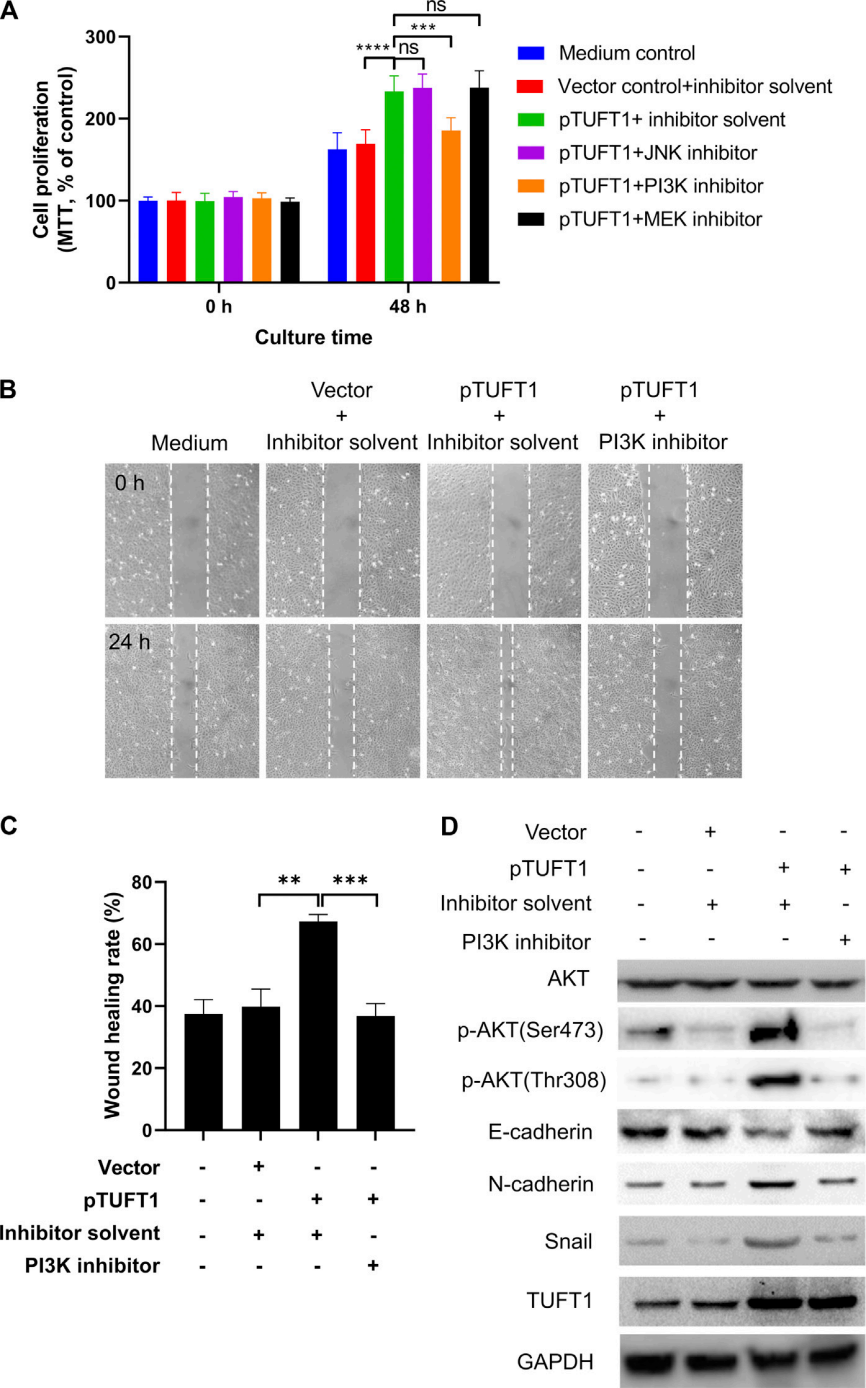
TUFT1 exerts oncogenic effects on RCC cells through PI3K/AKT signaling pathway. RCC cells were first mock-transfected or transfected with pTUFT1 and then treated with or without specific signaling pathway inhibitors. **(A)** cell proliferation was measured by MTT assay. Data shown are mean ± SD of three independent experiments. Ns, not statistically significant; ***, *p* < 0.001; ****, *p* < 0.0001. **(B, C)** Cell migration was determined by wound healing assay. One representative result is shown. **(D)** The expression of AKT, *p*-AKT, E-cadherin, N-cadherin, Snail and TUFT1 was determined by Western blot. One representative result is shown.

We next explored possible signaling pathways underlying TUFT1-mediated proliferation, migration and EMT progression of RCC cells. Canonical pathways involved in cancer progression (JNK, PI3K, and AKT pathways) were investigated using signaling pathway inhibition assay [33–35]. The effects of signaling pathway inhibitors on RCC cell proliferation was first determined. As shown in Figure 5A, the overexpression of TUFT1, in consistent with our previous data, promoted cell proliferation, and the addition of inhibitor solvent, or JNK and MEK inhibitors did not induce any apparent impact on the cell growth. Of note, the enhancement of RCC cell proliferation by TUFT1 overexpression was significantly suppressed by the introduction of PI3K inhibitor into the system, indicating PI3K pathway was involved in TUFT1-mediated RCC cell proliferation. Further experiments were performed to confirm whether this pathway was also involved in TUFT1-mediated migration and EMT progression of RCC cells. Similar to the proliferation assay, the migration assay showed that TUFT1 overexpression promoted RCC cell migration while the PI3K inhibitor counteracted at such enhancement (Figures 5B,C). Western blot results showed that TUFT1 overexpression did not increase the expression of AKT, a serine/threonine-specific protein kinase playing an important role in PI3K pathway but increased the phosphorylation of AKT (*p*-AKT) (Figure 5D). In consistent, the elevation of *p*-AKT, N-cadherin and Snail as well as the decrease of E-cadherin induced by TUFT1 overexpression were suppressed by the addition of PI3K pathway inhibitor (Figure 5D). These data here indicate that TUFT1 exerts oncogenic effects on RCC cells through PI3K/AKT signaling pathway.

Taken together, our current study has revealed that TUFT1 expression is increased in RCC tissue and cell lines both on the mRNA and protein levels. TUFT1 elevation in RCC can promote cancer cell proliferation, migration and EMT progression, *via* the PI3K/AKT signaling pathway.

## Discussion

Although remarkable advances have been made in the understanding of RCC tumorigenesis, the exact mechanism still remains elusive. TUFT1 is recently shown to be elevated in HCC and its elevation in expression promotes the progression of several types of cancers including HCC, breast cancer, thyroid carcinoma and osteosarcoma [18–20]. However, whether TUFT1 is also involved in the tumorigenesis of other tumors has not been investigated. In the current study, we have shown that TUFT1 expression was also increased in RCC. Furthermore, the increase of TUFT1 expression contributed to RCC progression by enhancing the proliferation, migration and EMT of RCC cells, through PI3K/AKT signaling pathway. The findings of our study not only advance the understanding of RCC pathogenesis, but also provide a potential diagnostic biomarker as well as treatment target.

TUFT1 is a phosphorylated glycoprotein that is initially discovered in tooth enamel and plays a role in dental enamel mineralization [16]. After synthesized in ameloblasts, TUFT1 is then secreted into the enamel matrix and accumulates at the dentin-enamel junction [36]. Later on, TUFT1 expression is detected in other non-mineralizing tissues and its functions seem to be multivariant and tissue dependent [37, 38]. It is until very recently that TUFT1 has been found to play a role in tumorigenesis [20, 39]. A previous study has shown that TUFT1 is elevated in HCC and such elevation is involved in HCC growth, migration and EMT. In addition, high TUFT1 expression is correlated with unfavorable clinical outcomes and poor prognosis [20]. In consistent with the findings in HCC, we herein have demonstrated that TUFT1 expression is also increased in RCC. In addition, our study has further revealed that TUFT1 can promote the proliferation, migration and EMT of RCC cells through PI3K/AKT signaling pathway.

RCC is not one entity but rather a heterogenous group of different types of cancers [2]. Each type has differences in genetic characteristics, histological features and clinical phenotypes [40]. Major RCC subtypes are clear cell RCC (ccRCC, approx. 75%), papillary RCC (approx. 15%) and chromophobe RCC (approx. 5%) [41, 42]. Both ccRCC and papillary RCC are believed to arise from the proximal tubular epithelium while chromophobe RCC and other rare subtypes are thought to arise from the distal nephron, probably from the collecting tubular epithelium [43]. In our current study, we have only checked the expression of TUFT1 in paired RCC cancer tissue and adjacent noncancerous tissue by western blot, showing that TUFT1 expression was increased in RCC tissue. However, cell specific TUFT1 expression remains unclear. In addition, we observed that TUFT1 expression was quite different between different patients. Although we do not know the reason regarding the TUFT1 expression difference, we suspect that TUFT1 expression is associated with RCC tumor stage. Of the 2 cases in our study showing lower TUFT1 expression than the rest of the cases, they are both in the early tumor stage (TNM stage T1 and T2, respectively). Similar difference in TUFT1 expression is also observed in HCC, which is reported to be associated with HCC tumor stage as well [20]. It would be interesting for further studies to investigate whether the difference in TUFT1 expression among different individuals are associated with disease progression and clinical features.

In cancer cells, many signaling pathways are abnormally activated to mediate malignant behaviors of the cells. Canonical cancer-related pathways include PI3K/AKT, JNK and MEK pathways, which increase cell proliferation and motility [44–46]. Here, we demonstrate that TUFT1 promotes AKT phosphorylation but not AKT expression in RCC cells while inhibition of PI3K pathway also suppresses the phosphorylation of AKT and RCC cell growth, migration and EMT, indicating TUFT1 exerts oncogenic functions in RCC through PI3K/AKT signaling pathway. Similarly, TUFT1 has also shown to enhance tumor cell growth and migration in HCC through the same signaling pathway [20]. However, in lung cancer cells, TUFT1 seems to enhance tumorigenesis through mTORC1 signaling pathway [39]. In addition, in breast cancer, TUFT1 promotes metastasis and chemoresistance through Rab5/Rac1 pathway [21]. These findings indicate that TUFT1 exerts oncogenic functions in different cancers, but the underlying mechanisms may differ. In addition, although it is beyond the scope of the current study, it would be warranted to investigate whether TUFT1 promotes tumor progression in other types of solid cancers and underlying mechanisms.

In the current study, we used signaling pathway inhibitors to determine the signaling pathway that is involved in TUFT1-enhanced RCC tumor progression. Our showed that inhibitor targeting PI3K, but not JNK or MEK pathways significantly inhibited RCC cell proliferation, migration and EMT, implying the involvement of PI3K pathway in this process. This was further confirmed by western blot that overexpression of TUFT1 could enhance the phosphorylation of Akt, a serine/threonine-specific protein kinase playing an important role in PI3K pathway. However, it must be noted that RCCs usually have high mTOR activity and inhibitors against the mTOR pathway are used to treat RCCs. Therefore, the PI3K pathway inhibitor might have affected other factors in RCC besides TUFT1. Another limitation of our current study is that all the experiments were mainly performed one cell line, A498, due to the limited research resource we could get. However, we have confirmed some of the key data using primary cancer tissue samples and another cell line 786-O.

Since TUFT1 promotes the growth, migration and EMT of RCC cells, it is possible that TUFT1 level may correlate with disease severity and/or patient survival. In fact, high TUFT1 expression is shown to correlate with poor clinical features and overall survival [20]. It is warranted to determine whether TUFT1 mRNA and/or protein level could be used as a biomarker for cancer diagnosis or stage classification. In addition, we and others have shown that inhibition of PI3K/AKT pathway could effectively suppress TUFT1-induced RCC cell proliferation and migration. It will also be interesting to investigate whether treatment strategies targeting this signaling pathway would present a novel approach for RCC treatment.

## Data Availability

The original contributions presented in the study are included in the article/Supplementary Material, further inquiries can be directed to the corresponding author.
