# Supplementary material for: Tuftelin 1 (TUFT1) Promotes the Proliferation and Migration of Renal Cell Carcinoma via PI3K/AKT Signaling Pathway
Source: Pathol Oncol Res. 2021 Apr 19;27:640936. doi: 10.3389/pore.2021.640936 (PMC8262214; doi:10.3389/pore.2021.640936)
Supplement: Supplementary file 1 [file DataSheet1.DOCX]

Supplementary Material

**Supplementary Table 1. The clinicopathological features of patients.**

| **Characteristics of patients** |  | **Number of patients** |
| --- | --- | --- |
| Age | <55 yr | 3 |
|  | >=55 yr | 7 |
| Histopathology | clear cell | 8 |
|  | papillary | 1 |
|  | chromophobe | 1 |
| Sex | Male | 7 |
|  | Female | 3 |
| TNM classification (T stage) | T1 | 3 |
|  | T2 | 4 |
|  | T3 | 2 |
|  | T4 | 1 |
| TNM classification (N stage) | N0 | 7 |
|  | N1 | 3 |
| TNM classification (M stage) | M0 | 9 |
|  | M1 | 1 |
| Previous systemic anticancer treatment | Yes | 0 |
|  | No | 10 |


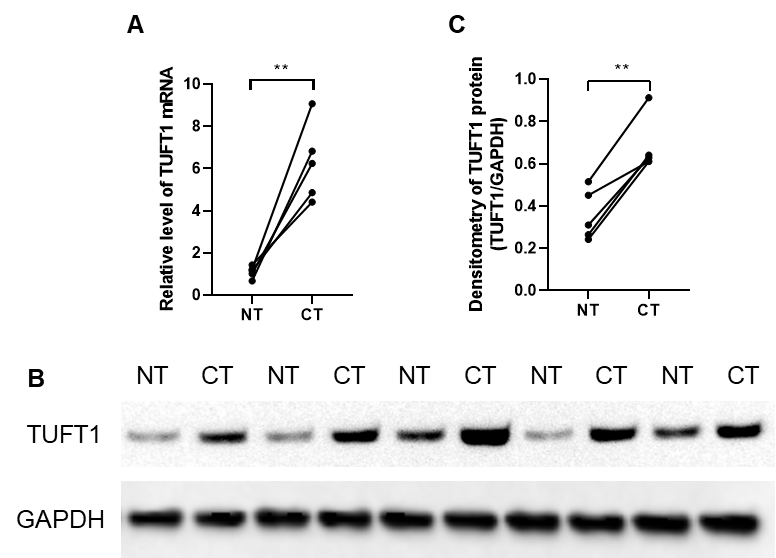


**Supplementary Figure 1.** TUFT1 expression is elevated in RCC tissue. (A-C) Cancerous tissue (CT) and adjacent noncancerous tissue (NT) were collected from RCC patients and (A) the mRNA and (B-C) protein level of TUFT1 were determined by RT-PCR and Western blot, respectively (n=5). (B) One representative result is shown. (C) Band intensity of (B) was semi-quantified by Image J. *, p < 0.05.


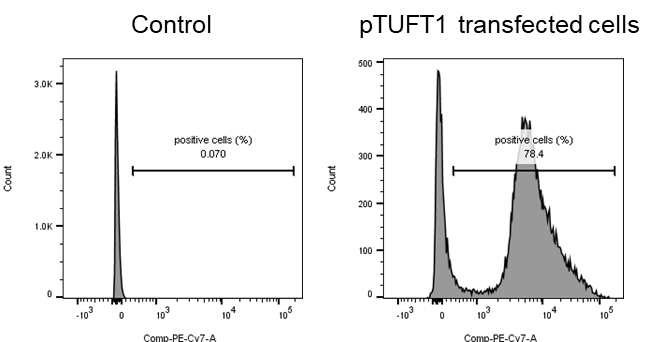


**Supplementary Figure 2.** A498 transfection efficiency by LIPO8000. A498 cells pre-seeded at 50-60% confluency were either mock-transfected or transfected with pTUFT1 using LIPO8000, according to the manufacturer’s instructions. When the cells formed a cell monolayer, cells were harvested and stained with rabbit anti-human TUFT1 followed by PE-Cy7 conjugated mouse anti-rabbit IgG. Following staining, cells were analyzed by flow cytometry. One representative data out of three is shown.
